# Combination antibiotics against *Pseudomonas aeruginosa*, representing common and rare cystic fibrosis strains from different Irish clinics

**DOI:** 10.1016/j.heliyon.2018.e00562

**Published:** 2018-03-08

**Authors:** Priya Kapoor, Philip Murphy

**Affiliations:** a Department of Clinical Microbiology, Adelaide and Meath hospital, Incorporating the National Children's hospital, Tallaght, Dublin, Republic of Ireland; b Department of Clinical Microbiology, School of Medicine, Trinity College Dublin, College Green, Republic of Ireland

**Keywords:** Microbiology, Infectious disease

## Abstract

**Objectives:**

To evaluate the effect of antibiotic combination therapy versus single therapy against cystic fibrosis strains of *Pseudomonas aeruginosa* identified as common and rare among patient groups in different Irish hospitals.

**Methods:**

This study compares the susceptibility profiles of *P. aeruginosa* isolates from different cystic fibrosis (CF) clinics in Ireland, collected from 2004–2005. Strains were recovered in small numbers and typed by pulsed-field gel electrophoresis. Five common clonal variants were identified in five different hospitals, described as ‘common strains’. A number of ‘rare strains’ associated with any single patient were also included in the study. Certain virulence factors were determined and *in vitro* assays such as minimum inhibitory concentrations (MIC) and biofilm inhibitory concentrations (BIC) were employed to assess potential synergistic effects of antipseudomonal antibiotic combination therapy.

**Results:**

There was no distinct virulence factors associated with clinical strains that were common in comparison to those that were rare. Antibiotic combination testing revealed the majority of combinations were similar to the activity of either antibiotic used as single agents. Tobramycin-ceftazidime was the most effective combination exhibiting synergistic interactions (FIC ≤ 0.5) against certain clinical isolates of *P. aeruginosa*.

**Conclusion:**

The efficacy of single antibiotics and synergistic interactions of antibiotic combinations were strain specific, irrespective of virulence characteristics of *P. aeruginosa*. Common clonal *P. aeruginosa* strains do not have distinct characteristics that possibly influence persistence in the chronic CF lung. Tobramycin-ceftazidime may be successful for controlling specific *P. aeruginosa* strains. Further studies on representative isolates are needed to support these results.

## 1 Introduction

Cystic fibrosis is an inherited autosomal recessive disorder that persists from mutations in the cystic fibrosis transmembrane regulator (CFTR) gene, enhancing viscous mucus production. Mucus prevents cilia beating on normal epithelia, enabling the growth of pathogens [1, 2]. *Pseudomonas aeruginosa* found in immunocompromised patients are one of the most prevalent species associated with CF respiratory tract infection. Chronic CF lung infection is caused by mucoid *P. aeruginosa* in which toxins and virulence factors are known to enhance colonisation and persistence in the respiratory tract leading to the formation of biofilms [3]. Biofilms develop due to bacterial cells becoming encased within an exopolysaccharide matrix [4], which may be attached to a surface or non-surface attached from the site of colonisation. They are difficult to eradicate with standard antibiotic therapy [5] as virulence factors vary across resistant strains [6].

Analysis of bacterial cultures from hospitals controls the outbreak of infection and reduces the risk of cross contamination [7]. Antibiotic susceptibility testing allows identification of the susceptibility profile of nosocomial pathogens [8]. The broth microdilution method enables biofilms to attach and develop to polystyrene pegs and treat with antimicrobial agents to determine susceptibility profiles [9]. The technique is rapid and allows numerous pathogens and agents to be screened simultaneously. Studies have optimised this technique for synergistic testing of antibiotic combinations. Combination therapy given as empirical treatment for *P. aeruginosa* is associated with a greater survival rate in comparison to monotherapy [10]. Combination of ceftolozane-tazobactam demonstrated higher *in vitro* activity against gram negative bacteria than each single agent [11]. Enhanced action of colistin and tobramycin against *P. aeruginosa* by targeting different components of the biofilm structure has also been reported [12].

In this study, we compared the activity of antibiotics singly and in combination against a cohort of CF *P. aeruginosa* isolates from a number of Irish hospitals. Pulsed-field gel electrophoresis established relationships between isolates and strains selected represented common clonal variants found in five different hospitals and rare isolates, unique to one patient. The objective was to determine whether antibiotic combinations are more advantageous than single antibiotic therapy and if virulence factors differ among the common and rare strains, influencing the response to antibiotic treatment.

## 2 Materials and methods

### 2.1 Selection of *Pseudomonas aeruginosa* strains

*P. aeruginosa* were obtained from different Irish hospitals from 2004–2005 (Table 1). Strains were recovered from cystic fibrosis patients chronically colonised with this bacterium, however, details such as age and sex was not available. All isolates were screened in Adelaide and Meath Hospital, incorporating the National Children's Hospital (AMNCH), Dublin, Ireland. Pigmentation and classification as mucoid or non-mucoid was recorded on tryptic soy agar (TSA) plates at 24 h, 35 °C (Table 2). Bacterial strains were stored at −80 °C, cultured on TSA, and typed using pulsed-field gel electrophoresis (PFGE). A difference of less than 4 banding patterns were described as related isolates and a difference of more than six bands was considered as unrelated isolates. PFGE analysis identified patient groups from five hospitals contracted a similar strain (common strains), unique to each hospital. Certain CF strains were non-typeable, while others used in this study were identified in no more than one patient (rare strains). Ten strains (5 common and 5 rare) were selected for testing. PA01 and ATCC 27853 were used as references. Strains were cultured in standard nutrient broth, (10 g peptone, 5 g sodium chloride, 10 g lab-lemco powder, pH 7.5 ± 0.2), Oxoid, for suitable bacterial growth.

**Table 1:** Sources of cystic fibrosis *P. aeruginosa* strains.

***P. aeruginosa* common strains**	**Source**
*CF strain 1*	Irish hospital 1
*CF strain 2*	Irish hospital 2
*CF strain 3*	Irish hospital 4
*CF strain 4*	Irish hospital 5
*CF strain 5*	Irish hospital 3

***P. aeruginosa* rare strains**	**Source**

*CF strain 6*	Irish hospital 2
*CF strain 7*	Irish hospital 2
*CF strain 8*	Irish hospital 3
*CF strain 9*	Irish hospital 4
*CF strain 10*	Irish hospital 6

**Reference strains**	**Source**

ATCC 27853	ATCC
PA01	ATCC

**Table 2:** Virulence characteristics of *P. aeruginosa* strains.

*P. aeruginosa* strain	Pigment	Mucoid (M)/Non-mucoid (NM)
*CF strain 1*	+	NM
*CF strain 2*	−	M
*CF strain 3*	−	M
*CF strain 4*	−	NM
*CF strain 5*	+	NM
*CF strain 6*	+	M
*CF strain 7*	+	M
*CF strain 8*	+	NM
*CF strain 9*	+	NM
*CF strain 10*	−	NM
PA01	+	NM
ATCC 27853	+	NM

### 2.2 Antibiotics

Four antibiotics, tobramycin (Sigma-aldrich), ceftazidime (Sigma-aldrich), meropenem (Sigma-aldrich), ciprofloxacin (Mylan, Inc.), were selected for testing based on effective antipseudomonal activity and were obtained from the department of pharmacy, AMNCH.

### 2.3 Determination of the minimum inhibitory concentration (MIC)

MIC values of antibiotics were determined by the microtitre method. Stock solutions of antibiotics were prepared and added to the bottom of a 96-well microtitre plate (Nunc Inc., Roskilde, Denmark). 100 μl of this solution was added to first well of the 96-well plate and serially diluted. 100 μl of an overnight culture of *P. aeruginosa* was added to each well at a final concentration of 5 × 10^5^ CFU/ml (colony-forming units per millilitre). The microtitre plates were incubated at 35 °C for 24 h and the MIC determined as the lowest concentration of antibiotic showing no visible bacterial growth.

### 2.4 *P. aeruginosa* biofilm growth assays

**(A)**. To assess the ability of *P. aeruginosa* isolates to form biofilms, a 5 × 10^5^ CFU/ml suspension of each strain was added to 96-well microtitre plates, covered with a transferable solid phase (TSP) pin lid containing pegs and subsequently incubated at 35 °C for 24 h and 72 h, respectively. TSP pin lids were washed three times in sterile water and left to dry at room temperature. Pegs were stained with 0.3% crystal violet for 30 minutes and rinsed again to remove any dye that did not bind to the cells. Cells stained with crystal violet were removed with a solution of 95% ethanol and 1% triton X-100 (1:1 ratio). Optical density (OD_630_) was measured in a microplate reader and biofilm growth was recorded at an OD_630_ ≥ 0.05 (Moskowitz *et al* [9]). This assay was conducted in triplicate. **(B).** Biofilm cell viability was measured by adding a 5 × 10^5^ CFU/ml suspension of each strain into the wells of a 96-well microtitre plate and incubating for 24 h and 72 h. Cells were collected, rinsed in sterile saline, serially diluted and plated onto TSA plates to determine CFU/ml.

### 2.5 Determination of biofilm inhibitory concentration (BIC)

*P. aeruginosa* isolates were grown according to biofilm growth assay **(A)**. 100 μl of each bacterial suspension was added to the wells of a 96-well round-bottom plate, representing 5 × 10^5^ CFU/ml of each isolate. TSP pin lid was placed into the microtitre plate and incubated overnight at 35 °C. Serial two-fold dilutions of antibiotics were added to a new 96-well plate to which the peg lid was transferred and incubated for 24 h at 35 °C. Lids containing pegs were stained with CV, rinsed and dried at room temperature for 30 minutes. To remove stained biofilm, the lid was placed into a new 96-well round-bottom microtitre plate containing a (1:1 ratio) solution of 95% ethanol and 1% triton X-100 and OD_630_ was measured. BIC was determined as the last well that had an OD_630_ ≥ 0.05. This assay was conducted in triplicate.

### 2.6 Determination of single versus combination antibiotic therapy

Bacterial strains were grown as described in the MIC and BIC assays. Two-fold serial dilutions of antibiotics were prepared at a concentration range 256–0.5 mg/L. Antibiotic combination testing involved a 1:1 ratio of two agents at the same concentration. Mature biofilms were incubated for 72 h with daily replenishment of nutrients and subsequently treated with antibiotics.

### 2.7 Fractional inhibitory concentration (FIC)

To determine the interactions that occurred between antibiotic combinations the FIC was calculated. The following definitions were used: ≤0.5 is synergistic, >0.5 to ≤1 is additive, >1 to ≤4 is indifferent and >4 is antagonistic, according to the FIC index.

### 2.8 Statistical analysis

Statistical analysis was carried out using PASW^R^ Statistics 18 - SPSS. One-way ANOVA was used to determine statistical variations of the experiments involving antibiotic combination therapy versus single antibiotic therapy. A *P* ≤ 0.05 value was considered statistically significant.

## 3 Results

### 3.1 Diversity of *P. aeruginosa* isolates grown as biofilms

Biofilm formation was quantified by **(A)** the ability to adhere to polystyrene pegs in 96-well plates quantified by crystal violet straining and **(B)** viable cell counts at 24 h and 72 h. The majority of *P. aeruginosa* clinical and reference strains showed a progressive increase in biofilm adherence from 24 h to 72 h on polystyrene pegs. *CF strain 6* produced significantly higher biofilm density than the other strains at 24 h and produced significantly more biofilm than ten out of twelve strains at 72 h (*P* ≤ 0.05, one-way ANOVA). *P. aeruginosa* strains, *CF strain 1*, *CF strain 6*, consistently formed strong biofilm, particularly at the 72 h time point. Growth rate of *Strain 7* was comparable at 24 h and 72 h.

**Fig. 1:**
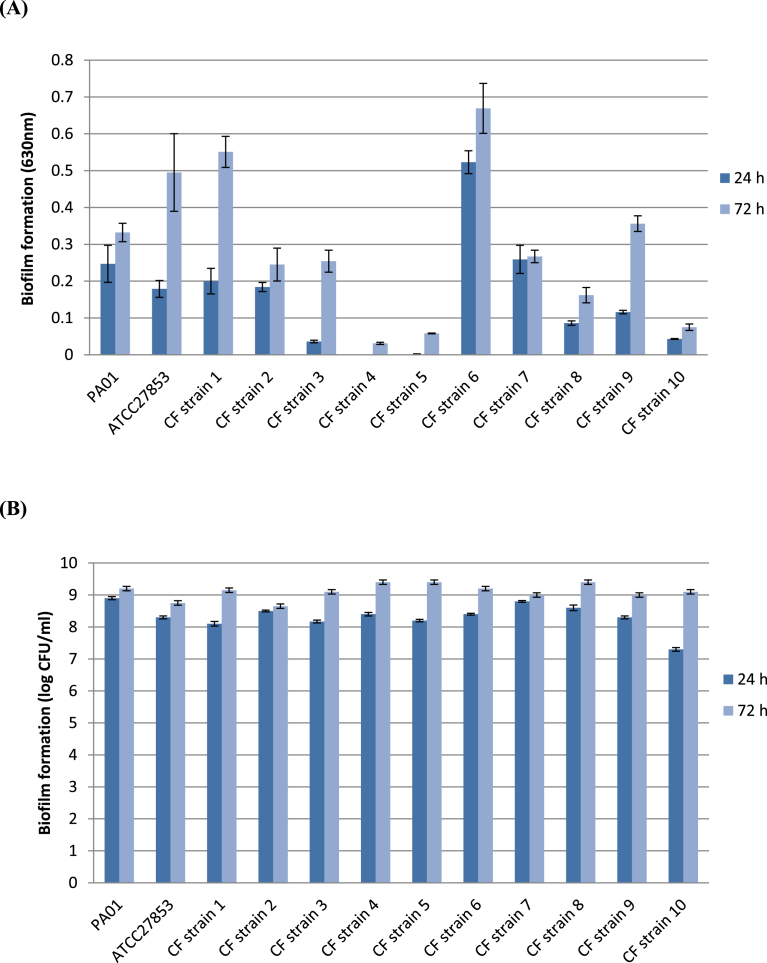
Biofilm formation of *P. aeruginosa* strains. Measurements of biomass were based on (A) OD_630_ on polystyrene pegs and (B) log CFU/ml. At 24 h and 72 h biofilm biomass was quantified for each strain. Data represent the mean ± standard deviation of three independent experiments.

The two models showed variation in the amount of biofilm formed by each strain (Fig. 1). A total of eight strains were characterised as strong biofilm formers at 24 h (OD_630_ ≥ 0.05) on pegs with a high increase in growth at 72 h. In contrast, four clinical strains (*CF strain 3*, *CF strain 4*, *CF strain* 5 and *CF strain 10*) which exhibited a low level of adherence to the pegs had high viable cell counts at 24 h and 72 h. *P. aeruginosa* strains are capable of producing biofilms of high cell density at 24 h and 72 h intervals even if the level of adherence to the pegs remains weak.

### 3.2 Single antibiotics against *P. aeruginosa*

CF *P. aeruginosa* isolates representing common strains (*CF strain 1*, *CF strain 2*), rare strains (*CF strain 6*, *CF strain 7*) and reference strains (PA01, ATCC 27853) were selected for antibiotic susceptibility testing. Strains were specifically selected from different hospitals and due to adequate biofilm-forming ability. Table 3 illustrates the susceptibilities of antibiotics tested singly against planktonic and biofilm grown isolates. Among the antibiotics selected ciprofloxacin was the most effective single agent against five out of the six *P. aeruginosa* isolates tested. Clinical isolate, *CF strain 2* displayed the greatest sensitivity to meropenem indicating antibiotics are displaying unique effects against the different strains. Ceftazidime treated cells exhibited much lower MIC than BIC values against the other *P. aeruginosa* strains indicating this agent can have reduced efficacy against certain biofilm-forming strains. Meropenem exhibited weak antibiofilm activity against three isolates (PA01, ATCC 27853, *CF strain 6*) in comparison to planktonic counterparts. Similarly, the growth pattern of *Strain 2* and *Strain 6* was weaker in response to tobramycin in biofilm form.

**Table 3:** Minimum inhibitory concentration (MIC mg/L) and biofilm inhibitory concentration (BIC mg/L) of single antibiotics against *P. aeruginosa*.

Antibiotic	PA01		ATCC 27853		*Strain 1*		*Strain 2*		*Strain 6*		*Strain 7*	
	MIC	BIC	MIC	BIC	MIC	BIC	MIC	BIC	MIC	BIC	MIC	BIC
Tobramycin	0.25	1	0.5	0.5	0.5	2	4	32	2	>256	0.25	0.25
Ceftazidime	4	128	2	2	1	128	1	128	8	>256	4	8
Meropenem	1	8	2	8	2	2	0.06	0.06	1	>256	1	1
Ciprofloxacin	0.06	0.06	0.06	0.25	0.125	0.125	1	1	0.5	1	0.25	0.25

### 3.3 Antibiotic combinations against *P. aeruginosa*

*P. aeruginosa* isolates were grown planktonically and as biofilms and treated with antipseudomonal antibiotic combinations (Table 4). Selected antibiotics ceftazidime and meropenem were examined in combination with tobramycin, since CF patients are frequently treated with this antibiotic. Ciprofloxacin was also tested in combination with the other antibiotics as it displayed strong inhibitory activity as a single agent against the majority of strains.

**Table 4:** Susceptibility of *P. aeruginosa* planktonic (MIC mg/L) and 24 h biofilm-grown isolates (BIC mg/L) to antibiotic combinations.

Antibiotic Combinations	PA01		ATCC 27853		*Strain 1*		*Strain 2*		*Strain 6*		*Strain 7*	
	MIC	BIC	MIC	BIC	MIC	BIC	MIC	BIC	MIC	BIC	MIC	BIC
Tobramycin-Ceftazidime	0.5	1	0.25	1	1	1	4	32	1	32	0.25	0.25
Tobramycin-Ciprofloxacin	0.25	0.25	0.25	0.25	0.25	0.25	2	4	1	>256	0.25	0.25
Tobramycin-Meropenem	1	2	0.25	0.5	0.25	0.5	0.25	0.25	0.5	>256	0.25	0.25
Ciprofloxacin-Ceftazidime	0.125	0.125	0.06	0.5	0.125	0.125	2	2	1	>256	0.25	0.25
Ciprofloxacin-Meropenem	0.125	0.125	0.25	0.25	0.125	0.125	0.5	1	1	>256	0.25	0.25

FIC results in Table 5 show the interactions that occurred between antibiotic combination therapy. Results revealed combinations tobramycin-ceftazidime and tobramycin-meropenem displayed synergistic activity (FIC ≤ 0.5) against the biofilm of *CF strain 1*. Tobramycin-ceftazidime also exhibited synergistic interactions against *CF strain 6* at an FIC ≤ 0.5. Interestingly, these antibiotic combinations did not have a similar affect against the same strains grown as planktonic isolates, indicating that these interactions may be specifically targeting the development of biofilms at certain growth stages. Antibiotic combinations tobramycin-ceftazidime and tobramycin-meropenem did not display synergistic interactions against the reference strains in planktonic or biofilm form. Ciprofloxacin which was an effective single agent displayed the same inhibitory activity in combination with other antipseudomonal antibiotics as ciprofloxacin monotherapy. Antagonistic interactions were observed with three antibiotic combinations: tobramycin-meropenem, ciprofloxacin-ceftazidime and ciprofloxacin-meropenem, against the biofilm of *CF strain 6*. Ciprofloxacin-meropenem also displayed antagonistic interactions against the biofilm of *CF strain 2* (FIC > 4).

**Table 5:** FIC of antibiotic combinations according to the FIC index.

FIC of Antibiotic Combinations	PA01		ATCC 27853		*Strain 1*		*Strain 2*		*Strain 6*		*Strain 7*	
	MIC	BIC	MIC	BIC	MIC	BIC	MIC	BIC	MIC	BIC	MIC	BIC
Tobramycin-Ceftazidime	2	1	0.6	2.5	3	**0.5**∗	5	1.5	0.65	**0.25**∗	1	1
Tobramycin-Ciprofloxacin	5	4	1.5	1.5	2.5	2	2.5	4	2.5	2	2	2
Tobramycin-Meropenem	1.4	2	0.6	1	0.6	**0.5**∗	4	4	0.75	275	1.25	1.25
Ciprofloxacin-Ceftazidime	2	2	1	2.25	1.1	1.1	4	2	2	257	1	1
Ciprofloxacin-Meropenem	2	2	1	1	1.06	1.06	8.5	17	3	8.5	1.25	1.25

∗ FIC value for synergy ≤0.5.

### 3.4 Effective antibiotic combinations against mature (72 h) *P. aeruginosa* biofilms

*P. aeruginosa* biofilms were grown to a level of maturity (72 h) and treated with tobramycin-ceftazidime and tobramycin-meropenem (Table 6). These combinations were selected as they displayed synergistic interactions against certain biofilms grown at 24 h. Two reference strains (PA01, ATCC 27853) and two clinical strains (*CF strain 1*, *CF strain 6*) were tested. *CF strain 1* was synergistically inhibited with tobramycin-ceftazidime at an FIC ≤ 0.5. Tobramycin-meropenem showed indifference under the same experimental conditions, indicating antibiotic interactions can be influenced by the age of the biofilm. Similarly, tobramycin-ceftazidime which acted synergistically against clinical isolate *CF strain 6* at 24 h did not display the same inhibitory effect at 72 h (FIC 2), shown in Table 7.

**Table 6:** Susceptibility of mature (72 h) *P. aeruginosa* biofilms (BIC mg/L) treated with effective single and combination antibiotics.

Antibiotic	PA01	ATCC 27853	*CF strain 1*	*CF strain 6*
Tobramycin	4	1	>256	>256
Ceftazidime	>256	>256	>256	>256
Meropenem	>256	>256	>256	>256
Tobramycin-Ceftazidime	4	1	64	>256
Tobramycin-Meropenem	4	2	>256	>256

**Table 7:** FIC of effective antibiotic combinations against mature (72 h) biofilms.

Antibiotic Combination	PA01	ATCC 27853	*CF strain 1*	*CF strain 6*
Tobramycin-Ceftazidime	1	1	0.5*	2
Tobramycin-Meropenem	1	2	2	2

∗ FIC value for synergy ≤0.5.

## 4 Discussion

It was interesting to observe all clinical strains had different virulence characteristics. There were no distinct characteristics found in the common strains that explain why they were recovered from a cohort of CF patients. Certain characteristics such as mucoidity can contribute to a more rapid impairment in lung function [13]. Studies have also described small colony variants (SCV) as highly adherent and commonly associated with biofilm formation in the CF lungs [14]. Biofilm growth analysis found species may not colonise the surfaces of pegs but may be depositing on the bottom of the microtitre wells, in accordance with the study by Smith *et al* [15]. This would explain the high biofilm density observed with the biofilm viability assay. Motility may play a role in biofilm formation through which cells need to find suitable places for biofilms to settle [16].

Antibiotic susceptibility testing enables the selection of suitable agents for treatment of CF bacterial infections [18]. A limited number of isolates were obtained for this study, however, results indicate CF isolates had unique responses to antibiotic treatment. *P. aeruginosa* biofilms were grown as described by Moskowitz *et al* [9] with slight modifications. This method does have limitations since biofilm structures are generally less developed than other assays. Results revealed biofilms were more resistant to antibiotics than planktonic cells, comparing favourably to the study by Aaron *et al* [18]. Ciprofloxacin was identified as a more effective single agent than tobramycin against strains grown planktonically and as biofilms. Previously, susceptibility testing of biofilms found fluoroquinolones such as ofloxacin and ciprofloxacin penetrate biofilm structures more effectively. Aminoglycosides such as tobramycin penetrate the biofilm structure at a slower rate than ciprofloxacin which may be allowing bacteria to acquire adaptive stress responses [19, 20].

Findings from the study found no synergistic antibiotic interactions against any planktonic strains. In addition, interactions of antibiotics did not rely on whether the isolate was mucoid or non-mucoid. *P. aeruginosa* strains that are more transmissible displayed a similar response to antibiotic treatment to less transmissible strains. A limited number of synergistic interactions were observed against biofilm-grown isolates, in particular tobramycin-ceftazidime. The mechanisms influencing this response remain inconclusive and warrant further study. Increased production of exopolysaccharide may be slowing the penetration of antibiotics through the biofilm matrix [21]. A previous study, found patients with lower respiratory tract infection treated with tobramycin-ceftazidime experienced a better clinical outcome that the group receiving ceftazidime monotherapy [22]. Findings from the study also suggest although tobramycin-ceftazidime may be effective for biofilms inhibition, changes that occur during biofilm maturation may reduce the efficacy of this treatment. This compares with the combination of colistin and tobramycin which was more effective *in vitro* than each single agent as different areas of the biofilm structure were targeted by each agent [12]. Antibiotics may display synergistic interactions against biofilms during the different stages of the biofilm developmental cycle [23]. Combined antibiotic treatment of colistin and ciprofloxacin allows biofilm cells to be treated during the different stages of metabolic activity [24]. In addition, biofilms treated with azithromycin and ceftazidime exhibited reduced bacterial adherence and virulence factor production [17].

The study contributes to the current knowledge of antibiotic combinations therapy for bacterial infection. There are a number of limitations with this study. Firstly, the collection of isolates remains small and it would be useful to analyse the susceptibility profiles of pathogens over a longer period. Analysis of strains that formed weaker biofilms would also be advised as such characteristics are frequently observed in clinical isolates. The reasons for transmissibility of strains remains unclear but may be due differences in infection control practices in the different hospitals. The key findings demonstrate synergistic activity of antibiotic combination therapy is limited and not reliant on the virulence factors of *P. aeruginosa*. Strains identified as common clonal variants among patient groups did not display any unique response to antibiotic treatment in comparison to strains that were rare. No single treatment ensures successful control of *P. aeruginosa* infection. Synergistic results of tobramycin-ceftazidime suggest this combination may be beneficial, however, further analysis of *P. aeruginosa* in a clinical setting is necessary to assess the interactions of this combination.

## Declarations

### Author contribution statement

Priya Kapoor: Conceived and designed the experiments; Performed the experiments; Analyzed and interpreted the data; Contributed reagents, materials, analysis tools or data; Wrote the paper.

Philip Murphy: Conceived and designed the experiments; Analyzed and interpreted the data; Contributed reagents, materials, analysis tools or data.

### Funding statement

This work was supported by the Irish Higher Education Authority, Centre of Applied Sciences and Health (CASH), Programme for Research in Third Level Institutes (PRTLI), Cycle 4.

### Competing interest statement

The authors declare no conflict of interest.

### Additional information

No additional information is available for this paper.
